# Eye movement patterns in complex tasks: Characteristics of ambient and focal processing

**DOI:** 10.1371/journal.pone.0277099

**Published:** 2022-11-09

**Authors:** Yuxuan Guo, Jens R. Helmert, Sven-Thomas Graupner, Sebastian Pannasch

**Affiliations:** 1 Institute of Psychology III, Engineering Psychology and Applied Cognitive Research, Technische Universität Dresden, Dresden, Germany; 2 Chair of Traffic and Transportation Psychology, Technische Universität Dresden, Dresden, Germany; 3 Centre for Tactile Internet with Human-in-the-Loop (CeTI), Technische Universität Dresden, Dresden, Germany; Indian Institute of Science, INDIA

## Abstract

Analyzing the time course of eye movements during scene viewing often indicates that people progress through two distinct modes of visual processing: an ambient mode, which is associated with overall spatial orientation in a scene, followed by a focal mode, which requires central vision of an object. However, the shifts between ambient and focal processing modes have mainly been identified relative to changes in the environment, such as relative to the onset of various visual stimuli but also following scene cuts or subjective event boundaries in dynamic stimuli. The results so far do not allow conclusions about the nature of the two processing mechanisms beyond the influence of externally triggered events. It remains unclear whether people shift back and forth from ambient to focal processing also based on internal triggers, such as switching between different tasks while no external event is given. The present study therefore investigated ambient to focal processing shifts in an active task solving paradigm. The Rubik’s Cube task introduced here is a multi-step task, which can be broken down into smaller sub-tasks that are performed serially. The time course of eye movements was analyzed at multiple levels of this Rubik’s Cube task, including when there were no external changes to the stimuli but when internal representations of the task were hypothesized to change (i.e., switching between different sub-tasks). Results suggest that initial ambient exploration is followed by a switch to more focal viewing across various levels of task processing with and without external changes to the stimuli. More importantly, the present findings suggest that ambient and focal eye movement characteristics might serve as a probe for the attentional state in task processing, which does not seem to be influenced by changes in task performance.

## Introduction

The existence of two distinguishable modes of visual processing has been a recurring theme in neurophysiological, neuropsychological, and psychophysical research over the past several decades (for review, see [1]); it is being suggested that these two modes of vision provide a powerful and unifying framework for understanding our pickup of visual information and our perception of the visual world. One of the well-known models of two modes of vision was proposed by Trevarthen [2], who studied the behavior of split-brain monkeys and concluded that there were two modes of vision in primates, one regulated by the subcortical system labeled "ambient" and one by the cortical system labeled "focal". Processing in ambient mode examines a large field of vision (in which the fovea plays a relatively minor role due to its small angular size), which helps build up a unified and stable space—a context—for action and recognition. Processing in focal mode, in contrast, is concentrated in the foveal projection, which focuses on high spatial frequency information and is sensitive to the slightest difference in position, orientation, luminance or hue. Ambient and focal modes of vision occur in a sequential fashion, as the ambient vision structures the spatial frame and defines the relative locations of objects so that an object/area of interest may be brought to full attention and analyzed by the focal vision. Bridgeman [3] later placed an emphasis on “motor” versus “cognitive” functions of ambient versus focal mode of vision: Visually guided behavior such as pointing accurately is mediated by the ambient vision, whereas perceptual judgement of a target’s direction or small motion is established by the focal vision.

In parallel with Trevarthen’s research, quite a few other studies also pointed to the existence of two modes of visual processing. Ungerleider and Mishkin [4] presented evidence that in the visual cortex of the monkey there were two separate pathways, one dorsal pathway dealt with object location and one ventral pathway dealt with object identification. This work laid the foundation for a recent model of Milner and Goodale [5], which has been developed partly through studies of neurological patients with local brain lesions [6,7]. Their interpretation of the functions of this later model emphasizes the sensorimotor character of dorsal processing, whereas conscious identification and recognition are attributed to ventral processing. Although the two visual mode dichotomies were stated in different terms, these findings are consistent with the general claim of differential processing by the two modes of vision and inspired numerous investigations of visually guided behavior and object recognition that reflect distinction between the two modes of vision [3,8–10].

Unraveling the neural mechanisms of two modes of vision arise the possibility of relating them to the major output of visual processing, that is, eye movements. The neural systems that govern eye movements are noteworthy as they form a network in the brain. Analysis of eye movements therefore provides a window into mechanisms in the brain, which advances our understanding of the role of eye movements within the active process of visual information sampling from the environment [11,12]. Real-life processing of visual information is based on sequences of fixations and saccades. During fixations, we hold the central foveal vision in a certain location to extract available information, whereas saccades relocate our overt attention from one point of interest in the visual world to another. Several eye movement studies of scene perception [13–16] demonstrated systematic variations in fixation durations and saccadic amplitudes during visual processing. These systematic variations are of particular interest as they may serve as diagnostic characteristics of two modes of visual processing [14,15,17–20].

Antes [16] was one of the first reporting a variation in fixations and saccades as subjects viewed complex pictures: Mean fixation duration increased while at the same time mean saccade amplitude decreased as viewing time progressed. Based on Karpov et al. [21], this pattern of visual exploration was explained as a continuous change from surveying informative aspects of the entire picture to the close examination of details. Later, Unema et al. [15] reported systematic variations within fixation durations and saccade amplitudes over the course of visual information processing. In their study, large saccades and short fixations were observed during the first few seconds of viewing a scene, whereas after a few seconds of viewing, relatively smaller saccades and longer fixations were measured. The authors attributed this effect to the shift from ambient to focal processing mode in accordance with Trevarthen [2]. The large saccades and short fixations during the initial viewing interval represented ambient processing mode, allowing for gathering information from peripheral vision. In contrast, the smaller saccades and longer fixations during the later viewing interval were explained as focal processing mode, allowing for extracting detailed information from central vision. In line with former studies, Pannasch et al. [14] yielded further insights into the relationship between the ambient/focal visual processing and eye movement patterns. In their study, a shifting relationship between fixation durations and saccade amplitudes was observed during different natural viewing conditions across a period of six seconds: After the onset of a scene, fixation durations increased while saccade amplitudes decreased over the course of scene inspection. The obtained differences in eye movement patterns between the first two seconds and the last two seconds of inspection time were interpreted as a shift from ambient to focal mode during visual processing. Pannasch et al. [14] suggested that the initial ambient gaze patterns served mainly for a spatial orientation in the environment [1], while the later focal viewing behavior enabled a more in-depth and object-centered processing. This ambient to focal processing behavior was found to be stable and was not influenced by factors which are known to affect fixations and saccades (e.g., object density or mood manipulation). A further experiment by Fischer et al. [19] demonstrated characteristic changes during free scene viewing in oculomotor and electrophysiological data: The observed increase in fixation durations with the decrease in saccade amplitudes over the time course of scene viewing, together with the changes in the respective components of the event-related potentials (i.e., C1, N1, and P2), indicated a shifting balance between the underlying two attentional mechanisms.

Evidence on ambient and focal processing mechanisms is not only based on the presentation of static scenes, where the onset of a new scene serves as trigger eliciting ambient to focal behavior. Research on visual processing of dynamic stimuli provides further evidence that eye movement patterns display a similar shift during viewing of dynamic stimuli. Pannasch [22] analyzed eye movement patterns of subjects who viewed video sequences. The author compared viewing behavior before and after abrupt scene cuts in the video sequences and observed an interplay between ambient and focal processing modes around these scene cuts. Subjects displayed longer fixations and shorter saccades before the scene cuts and then shifted to shorter fixations and longer saccades after the scene cuts, suggesting a shift from focal to ambient processing after an interruption of the dynamic information flow that caused by abrupt scene cuts. Furthermore, Eisenberg and Zacks [23] investigated ambient and focal visual processing during viewing of continuous dynamic scenes, i.e., movies of an actor completing an everyday activity. The authors observed shifts from focal to ambient processing (fixation durations decreased and saccade amplitudes increased) during the few seconds following subjective event boundaries, where activity features changed and the activity became unpredictable.

The design of these experiments, however, does not allow conclusions about the nature of ambient and focal processing beyond the influence of externally triggered events. For instance, it remains unclear whether people shift back and forth from ambient to focal processing also based on internal triggers, such as shifts within and across the processing of tasks while no external event is given. Correspondingly, an extended approach for examining the temporal signatures of the two processing modes is needed to provide further insight into what role ambient to focal shifts could play in task processing.

Apparently, the methodological question at hand is: What demarcates early (i.e., ambient) and late (i.e., focal) phases in a continuous task setting? Apart from the starting point of the task which provides a clear onset of processing, there are no strict external cut-points during which a particular processing behavior could be expected. One way to determine cut-points in a continuous task at which ambient processing might be initiated, is to break down the task into sub-tasks. The different sub-tasks are performed in sequence and serve as basic components for completing the macro task; switching between different sub-tasks parses the ongoing task progress into individual sub-tasks processes. Because the internal representations of the task are hypothesized to change when switching from one sub-task to another, it is likely that subjects will initiate ambient gaze patterns at these points for exploratory processing of the new sub-task. After this exploratory (ambient) processing, subjects will likely switch to focal gaze patterns for an in-depth processing of the details in order to complete the sub-task.

**Fig 1:**
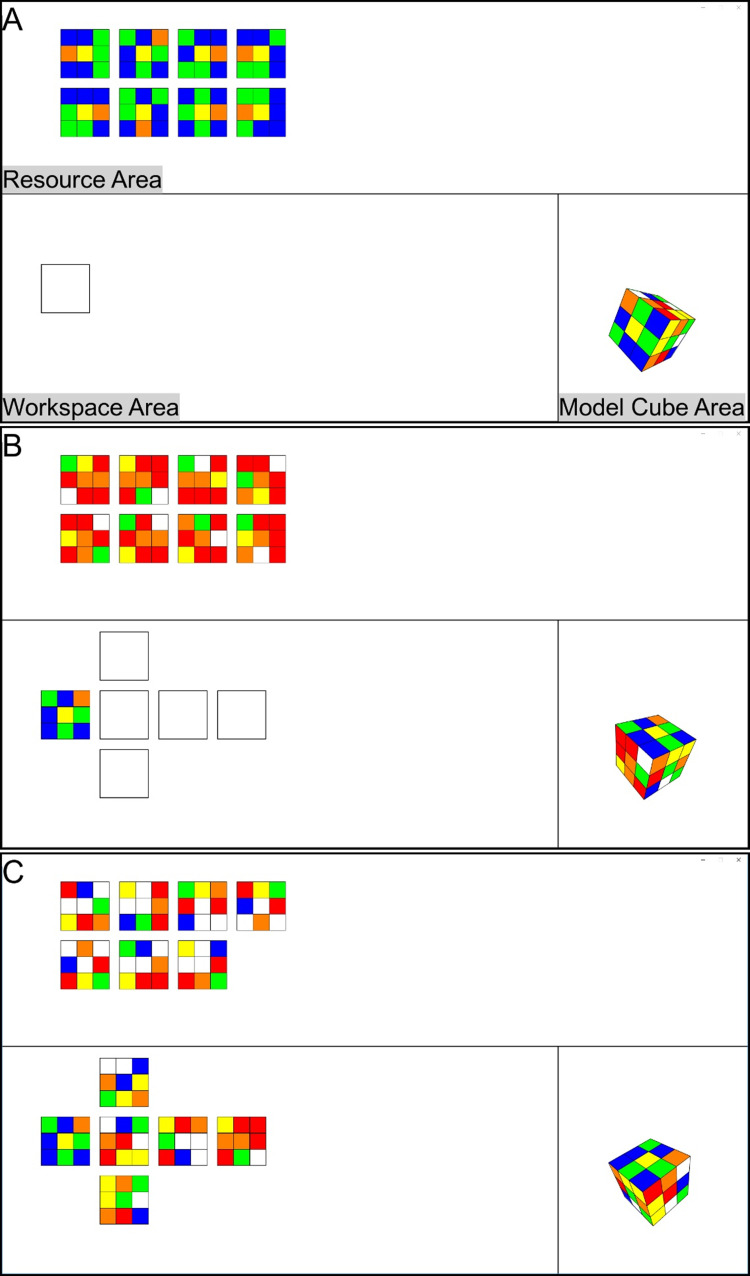
Examples of the Rubik’s Cube task scenario. The RC task scenario contained the model cube area, resource area, and workspace area. During the experiment, the areas contained no text label. In one RC task, the subject constructed one 2D copy. Completing one task required six target faces to be serially selected and moved from the resource to the workspace area. In each task, the position in workspace area where to place the first target face was framed (A). After the subject successfully completed the first face, a new set of resource faces appeared in the resource area and the other five positions appeared in the workspace area at the same time (B). The position in which every target face should be placed depended on how the 3D model cube faces connected to each other (spatial logic). When all positions were finished (C), the task was completed, the start of a new task followed up.

Therefore, the present study employed a paradigm where multiple steps had to be performed to solve the task. This task design allows to investigate ambient and focal processing mechanisms in the context of task processing. We developed the Rubik’s Cube task (henceforth RC task) that required subjects to duplicate a three-dimensional (3D) view model Rubik’s Cube into a two-dimensional (2D) pattern, following the spatial logic of the model cube configuration. The hierarchical structure of this task allows to identify smaller sub-tasks. The *task level* on top of the hierarchy represents the duplication of one 3D model cube, i.e., the subject constructs a complete 2D copy of one 3D model cube. The 2D copy consists of six faces (see bottom panels of Fig 1C with the 2D copy on the left and the 3D model cube on the right). Below the task level is the *face level*, where one of the six faces of the 3D model cube needs to be selected and allocated. Completing the face level requires subjects to gather and integrate information from three different areas at the *area level*: The *model cube area* displays the 3D model Rubik’s Cube, the *resource area* presents eight different resource faces (seven random faces and one face that depicts one face of the 3D model cube), and the *workspace area* where the selected face needs to be placed in order to build the 2D copy (see Fig 1A). In order to accomplish the RC task, subjects needed to be engaged at the area level, the face level, and the task level (example of a RC task: see Supporting Information, S1 Video).

Analyzing eye movement behavior at the task level is similar to previous work [14,15], where the onset of the task brings up a new visual environment (similar to a full display change). At the face level, however, only a part of the task environment changes: After finishing the first face, only new faces are presented in the resource area, while the 3D model cube and the completed face in the workspace area remain unchanged. The most intriguing question for the present work is whether ambient to focal processing behavior can be identified at the area level, i.e., when subjects switch between different areas of the task scenario. At the area level, there is no external change to the task environment, subjects shift their gaze and their visual attention in a self-paced manner.

In the study reported here, we investigated the time course of eye movements at multiple levels of a RC task, i.e., the task level, the face level, and the area level. In previous studies [14,15,19,23], the ambient to focal visual processing has been found relative to changes in the environment, such as relative to the onset of various visual stimuli but also following subjective event boundaries in dynamic stimuli. Based on these findings, we anticipated the occurrence of ambient to focal processing behavior following external changes to the stimuli during RC task solving. Specifically, we hypothesized that following the onset of a new task (task level) or the appearance of new resource faces (face level), fixation durations would increase while saccade amplitudes would decrease with time, indicating shifts from ambient to focal processing. Furthermore, based on the assumption that internal triggers would have a similar effect on attentional processing as external events, we predicted ambient to focal behavior to occur when switching between different areas of the task scenario. The various areas contain distinct information, i.e., the model to copy is displayed in the model cube area, the face to select is available in the resource area, and the correct position that needs to be found and fulfilled locates in the workspace area. Consequently, different sub-tasks were attributed to each of the areas. The model cube area mostly serves for comparison and information gathering, which could be achieved by rotating the 3D model cube. Visual searching for the appropriate face happens in the resource area and arranging the face into correct position takes place in the workspace area (picking and dragging the selected face from the resource area to the workspace area need to be performed). When subjects switched from one area to another, the internal representations of the RC task changed (e.g., a switch from rotating the 3D model cube to visual search of the resource area). We therefore hypothesized that after switching from one area to another, an increase in fixation durations together with a decrease in saccade amplitudes would be observed over the course of processing. Alternatively, one could only accept that distinct sub-tasks require various forms of processing, but this must not necessarily lead to the ambient to focal behavior when switching between the areas. Assuming that ambient processing is a useful strategy for gathering information about changes in the environment, evidence for the ambient to focal behavior should only be found at the task and the face level but not when switching between the areas. For the latter, one would expect a rather stable focal processing behavior, i.e., long fixation durations with short saccade amplitudes. However, regardless of the ambient to focal behavior, we also expected differences in oculomotor measures at the area level. As distinct sub-task processes took place in respective areas, eye movements in each area were expected to be more adaptive to the sub-task at hand. Therefore, differences in fixation durations and saccade amplitudes were anticipated across the three areas. While distinct eye movement characteristics (fixation durations/saccade amplitudes) were assumed at the area level, the main interest was in looking for the presence or absence of the ambient to focal gaze patterns across different areas.

Furthermore, it is worth noting that there is more than one way to solve the RC task, and the performance of the task (i.e., task solving time and accuracy) is likely to become mastered to certain extent over time. Based on the assumption that ambient to focal processing behavior represents a basic attentional mechanism, we expected a stable behavior over time that would be not altered by improvements in task solving proficiency.

## Methods

### Subjects

Volunteers were recruited from the Technische Universität Dresden subject pool. Subjects received either course credit or €10 payment for participation in a single experimental session (~ 90 min). All subjects had normal or corrected to normal vision. Subjects were informed of the purpose of the study before signing their consent form. Fifty-five subjects completed the study. Data from 55 subjects were included in the analyses reported here (age range: 18–42; mean age: 25; 35 females and 20 males). The study was conducted in line with the declaration of Helsinki and was approved by the local ethics committee of Technische Universität Dresden (ethic code: EK600122019).

### Apparatus

Subjects were seated individually in a dimly illuminated, sound-attenuated room. Eye movements were sampled monocularly at 500 Hz using the EyeLink 1000+ eye-tracking system (SR Research, Ontario, Canada) with online detection of saccades and fixations and a spatial accuracy < 0.5°. Saccades and fixations were identified using the saccade detection algorithm supplied by SR Research: Saccades were identified by deflections in eye position in excess of 0.1°, with a minimum velocity of 30°/s and a minimum acceleration of 8000°/s^2^, maintained for at least 4 ms.

For optimal eye-tracking, the eye tracker was run in the head-stabilized mode, i.e., subjects positioned their head on a chin-and-forehead rest. The eye-tracking camera was positioned 60 cm from the subject’s eyes. The RC task was displayed on an ASUS full-HD monitor (screen resolution: 1920 by 1080 pixels, screen size: 53 by 30 cm, viewing distance of 90 cm from the subject’s eyes, viewing angle of 32.81° horizontally and 18.92° vertically). Before starting the trial, a 9-point calibration and validation were performed and repeated if the error in any fixation point exceeded 1° or if the average error for all fixation points was more than 0.5°. During the experiment, if subjects required a break, the experiment would be paused and they could take a 5 min break. After the break, the calibration and validation procedure was performed again before resuming the experiment. The experiment always resumed with the start of a new RC task.

### Stimuli

During the experiment, subjects were instructed to duplicate a 3D model Rubik’s Cube into a 2D pattern. The task scenario consisted of three areas: model cube area, resource area, and workspace area (see Fig 1). The spatial dimensions (the x- and y-dimension) of the respective areas were classified as follows: the model cube area located in right bottom part (x = 1420–1920, y = 500–1080, 290.000 px^2^), the resource area located in the upper part (x = 0–1920, y = 0–500, 960.000 px^2^), the workspace area located in the left bottom part (x = 0–1420, y = 500–1080, 823.600 px^2^) of the task scenario. In the model cube area, a 3D view Rubik’s Cube configuration was displayed, which could be freely rotated by using the mouse. The resource area contained a set of eight different 2D faces. Seven of the resource faces were random arrangements, and only one target face corresponded to a face of the 3D model cube in the model cube area. The workspace area contained six framed positions for placing the selected faces from the resource area. The faces could be moved from the resource to the workspace area via drag and drop by using the mouse.

### Procedure

In order to solve the RC task, subjects had to (i) gather the necessary information about copying-details by rotating the 3D model cube in the model cube area, (ii) search the resource area for the target face that is needed for constructing the 2D copy, and (iii) guide the mouse to move the target face identified during the search phase from the resource area to the correct position in the workspace area. Subjects repeated the steps (i), (ii), and (iii) of individual order until the whole 2D copy was completed. To ensure subjects clearly understand the rule of the RC task, the experiment started with one practice task. In one experimental session, the subject needed to solve twenty RC tasks.

Subjects were instructed to duplicate the 3D model cube into a 2D copy as quickly as possible without making errors. In case of an error, i.e., selecting the wrong target face or dragging the selected face into a wrong position in the workspace, the selected face was always automatically restored to its former location in the resource area. The decisions on which resource face to select and where to place the face were made by subjects. Subjects were free to adopt any strategy.

After the experimental session, subjects had to complete a retrospective query where they should describe the strategies they used to solve the RC task and how the strategies changed over the course of the experiment.

### Data analyses

All data analyses were carried out using MATLAB R2021b and R. Results were analyzed in terms of task performances, task solving strategies, and eye movement patterns across the three levels of the RC task (task level, face level, and area level). Gaze and behavioral data of uncompleted tasks, i.e., when a subject took a break before completing a 2D copy construction, were discarded from data analyses.

Task performance analyses focused on the solving time and accuracy of each task. The measure of solving time started with the appearance of a new 3D model cube and terminated when the subject completed the 2D copy construction in the workspace area. Meanwhile, the errors subjects made during building every 2D copy were counted throughout the experiment. Additionally, subjects’ answers to the two retrospective questions were reviewed and analyzed in terms of the strategy features and using frequencies.

Eye movement analyses focused on temporal changes in fixation durations/saccade amplitudes to identify characteristics of ambient and focal processing modes. Raw eye movement data were pre-processed before the analyses. Fixations/saccades that occurred outside the presentation screen or contained blinks were excluded from analyses. Additionally, fixations less than 100 ms were removed if they occurred on either side of a blink. Fixation durations were trimmed to remove all fixations that were less than 40 ms. This removed 9.8% of raw eye-tracking data and left a total of 963,426 fixations and saccades as the database for further processes (complete data are available as Supporting Information, S1 Data).

In previous studies [14,15,20,23], the shifts between ambient and focal processing have been identified relative to changes in the environment. To test the hypothesis that fixation durations/saccade amplitudes would show phasic changes during processing of external changes in the task environment, we analyzed the time course of fixation durations/saccade amplitudes following the onset of a new task (task level) or the appearance of new resource faces (face level).

To investigate whether the ambient to focal processing would also occur when there were no external changes in the task environment but when internal representations of the task were hypothesized to change (i.e., switching between different sub-tasks), we analyzed and compared the time course of eye movements when switching between different areas of the task scenario (processing at the area level). We defined a temporal window of observation for eye movements in resource, workspace, and model cube area, respectively. The temporal window started when the subject’s eyes entered one area and terminated when the eyes left the area. However, to complete the task, subjects had to move the target face from the resource area to the workspace area. During visually tracking of the moving target face, subjects might initiate voluntarily smooth pursuit eye movements which allow maintaining the image of the moving object on the fovea and correct any velocity error between eye and moving target [24–26]. To prevent potential influences of smooth pursuit eye movements on our eye-tracking measurement, the last fixation and saccade in every temporal window of observation before leaving an area were excluded from analyses. Additionally, in order to ensure that subjects conducted enough gaze activity for processing information and dynamic changes in the area, episodes containing less than four fixations were excluded from analyses. The above pre-processing steps further removed 55.6% of the database for the analyses at the area level.

**Fig 2:**
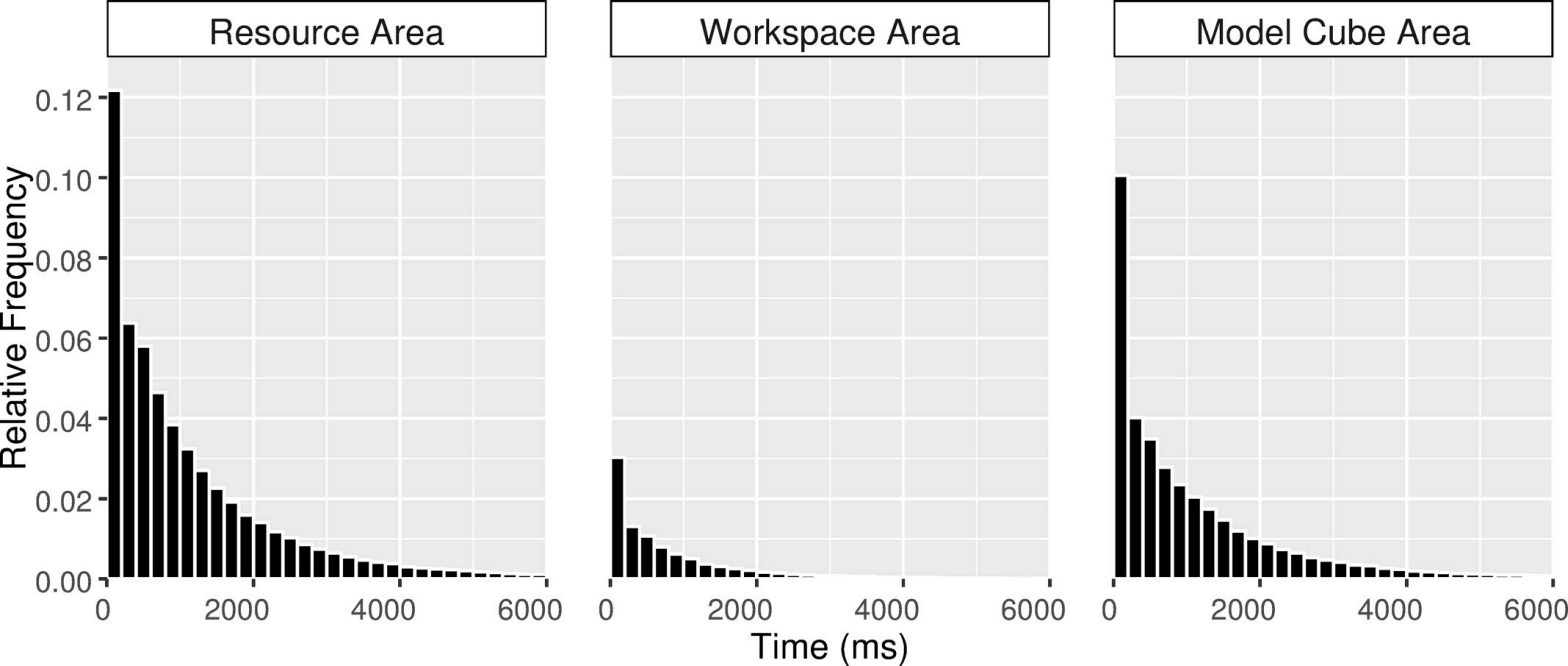
Relative frequency histograms of fixations for resource, workspace, and model cube areas. Relative Frequency histograms show the distributions of fixations in terms of time and areas. The temporal window of observations is divided into equally sized time bins. The shaded bars indicate time bins of 200 ms. Fixations were assigned into each of bins based on their starting points.

In order to determine a time interval within which ambient to focal behavior could be identified and compared across the different levels (task level, face level, and area level), we investigated how long subjects generally looked continuously in each of the areas (see Fig 2). The pooled relative frequency histograms of fixations revealed differences in the quantity of fixations across the three areas: most of fixations were allocated to the resource area, whereas the workspace area contained less fixations than the other two areas. More importantly, in the workspace area, fixations were only present within the first 3000 ms of entering the area. Therefore, we defined a time interval of 3000 ms for measuring ambient and focal gaze patterns at multiple levels of a RC task. Table 1 shows the numbers of fixations and saccades that remained for each of the analyses (at the task level, the face level, and the area level) within the time interval of 3000 ms subsequent to the above-described data pre-processing procedures. Furthermore, to assess how frequently subjects transited from one area to another, mean numbers of instances per subject within 3000 ms interval were identified for respective analyses (see Table 1). For discussion of an alternative method for data pre-processing, see Supporting Information, S1 Text.

**Table 1:** Numbers of fixations and saccades as well as mean numbers of instances per subjects identified for respective analyses.

	Fixations and Saccades		Instances per subject
	Number	Percentage	Mean (SD)
Database	963,426	100%	
Task level	18,044	1.9%	20
Face level	84,610	8.8%	100
Area level: Resource area	208,320	21.6%	337.35 (119.15)
Area level: Workspace area	37,972	3.9%	81.62 (56.29)
Area level: Model cube area	139,836	14.5%	288.53 (133.63)

Numbers in parentheses are standard deviations.

To investigate fixations and saccades along the time course across the different levels, the fixation durations and saccade amplitudes were binned into 200 ms time bins [14,15], and only appeared in the bins where they started. According to previous eye-tracking studies [27–30], fixation durations and saccade amplitudes were expected to reveal a positively skewed distribution where the median is less affected by extreme values and provides a more robust measure of central tendency than the mean. Subsequently, medians of fixation durations/saccade amplitudes commencing 200 ms bins were computed. Furthermore, medians of subjects’ fixation durations/saccade amplitudes per 200 ms bin were subjected to repeated measures analyses of variance (rm-ANOVA) or linear mixed-effects models for statistical testing. In the rm-ANOVAs, when the sphericity assumption was violated, the Greenhouse-Geisser correction was applied. Under this circumstance, epsilon values and corrected results are reported [31]. In addition, partial eta squared values are reported to demonstrate the potential practical significance of differences.

## Results

### Task performance

**Fig 3:**
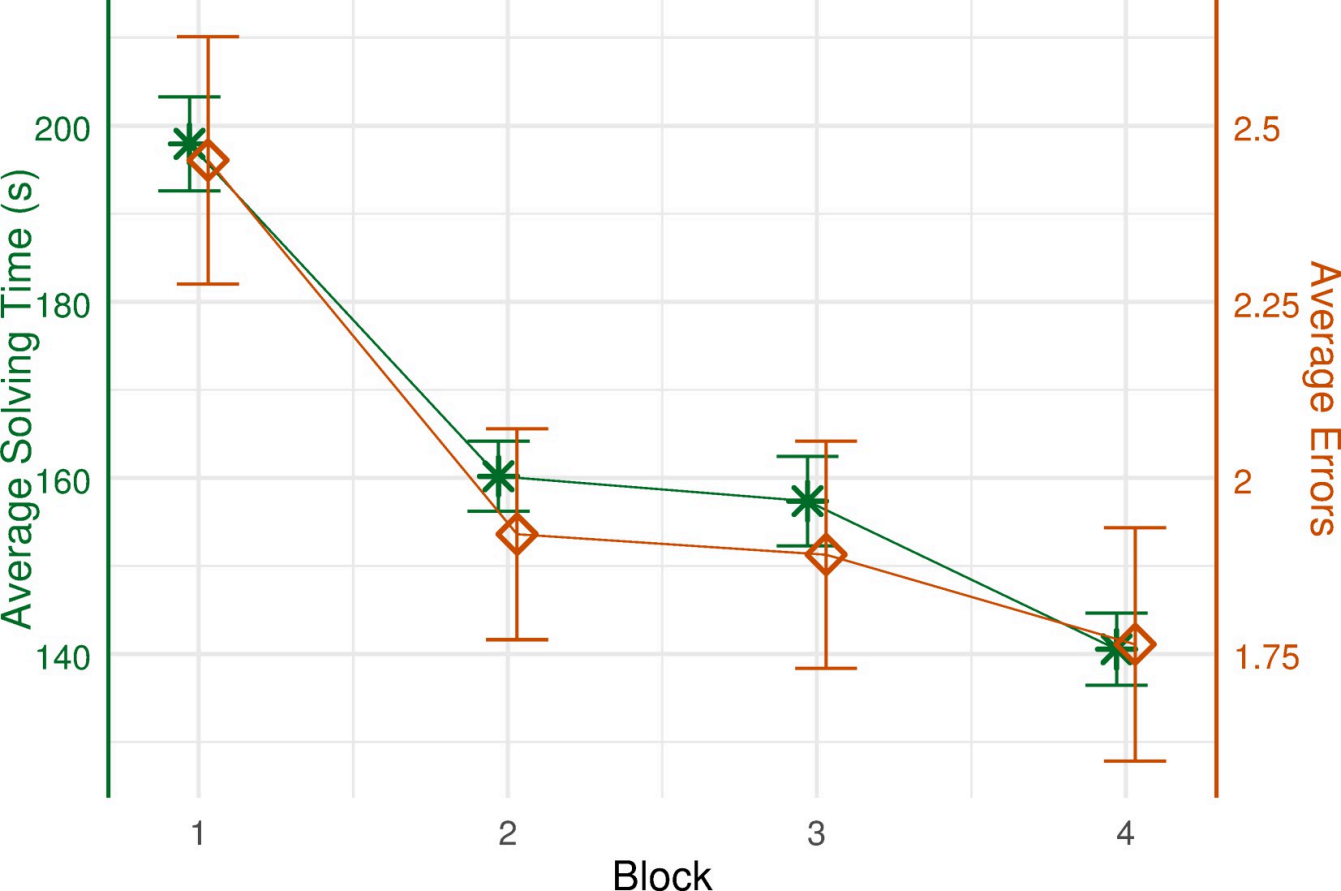
Average solving time and errors over four block. Average solving time and average errors per task during every block were calculated. Error bars represent ±1 standard error.

When performing the task, many subjects made errors. For instance, subjects selected the wrong face from the resource area or placed the selected face into a wrong position in the workspace area. Such errors and solving time of each task were examined throughout the experiment. To better assess subjects’ task performance over time, we clustered the twenty tasks evenly into four blocks of five tasks, respectively. The average task solving time and errors per blocks were calculated (see Fig 3). Decreases in both solving time (*Ms* = 197.93 vs. 160.18 vs. 157.36 vs. 140.54 s) and errors (*Ms* = 2.45 vs. 1.92 vs. 1.89 vs. 1.76) were observed, suggesting that subjects improved their task performance over time.

The rm-ANOVAs were conducted to compare the solving time and errors across four blocks, respectively. A significant effect of block on solving time was reported, *F*(2.4, 129.45) = 31.023, *p* < 0.001, *ε* = 0.799, *η2* = 0.365. Post hoc pairwise t-test comparisons with Bonferroni correction showed that subjects required significantly less time for solving one task during the last block than during the early three blocks (all *ps* < 0.01). Moreover, a significant effect of block on task errors was found, *F*(2.6, 140.34) = 4.916, *p* = 0.004, *ε* = 0.866, *η2* = 0.083. Post hoc pairwise t-test comparisons with Bonferroni correction revealed that subjects made significantly fewer errors during the later three blocks than during the first block (all *ps* < 0.05).

### Ambient to focal eye movement behavior

**Fig 4:**
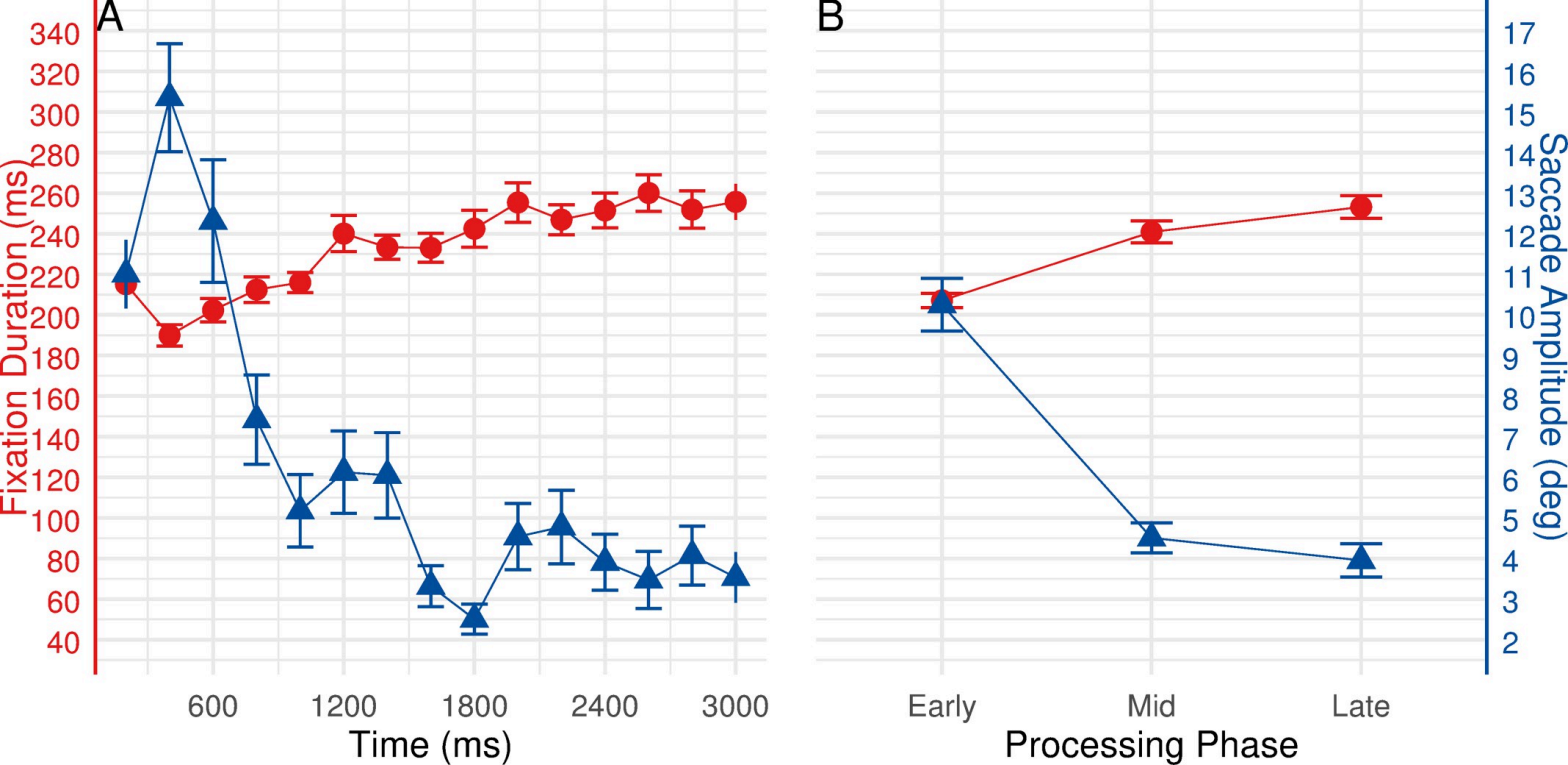
Eye movement behavior relative to the onset of new task. Left panel (A) shows fixation durations (red line) and saccade amplitudes (blue line) over time. Right panel (B) shows estimated marginal means (estimated under the rm-ANOVAs) for fixation durations and saccade amplitudes during early, mid, and late phases. Error bars represent ±1 standard error.

Fixation durations and saccade amplitudes at the task level. Analyses of fixation durations and saccade amplitudes at the task level over a 3000 ms timescale (see Fig 4) revealed the predicted behavior: As shown in Fig 4A, following the onset of the task, fixation durations increased while saccade amplitudes decreased over time.

To better identify the temporal signatures of ambient and focal processing modes, we compared eye movement patterns among early, mid, and late processing phases (see Fig 4B). The three processing phases were classified as follows: The early processing phase comprises the first 1000 ms of processing time, the period from 1000 to 2000 ms is considered as mid processing phase, whereas the late phase is defined as the period of 2000 to 3000 ms of processing time. The rm-ANOVAs were conducted to compare median values of fixation durations and saccade amplitudes across the three processing phases, respectively.

Analysis revealed significant changes of fixation durations over the three processing phases, *F*(1.79, 96.75) = 66.596, *p* < 0.001, *ε* = 0.896, *η2* = 0.552. Post hoc pairwise t-test comparisons with Bonferroni correction confirmed that fixation durations significantly increased throughout the three processing phases (all *ps* < 0.01, see Fig 4B): early phase (*M* = 207.15, *SE* = 3.52), mid phase (*M* = 240.85, *SE* = 5.42), and late phase (*M* = 253.21, *SE* = 5.61). Meanwhile, significant changes between the processing phases for saccade amplitudes were found, *F*(1.8, 97.21) = 67.113, *p* < 0.001, *ε* = 0.9, *η2* = 0.554. Follow-up post hoc pairwise t-test comparisons with Bonferroni correction revealed a significant decrease in saccade amplitudes from early to mid, as well as from the early to late phase (both *ps* < 0.001, see Fig 4B): early phase (*M* = 10.25, *SE* = 0.65), mid phase (*M* = 4.51, *SE* = 0.37), and late phase (*M* = 3.95, *SE* = 0.41).

### Fixation durations and saccade amplitudes at the face level

**Fig 5:**
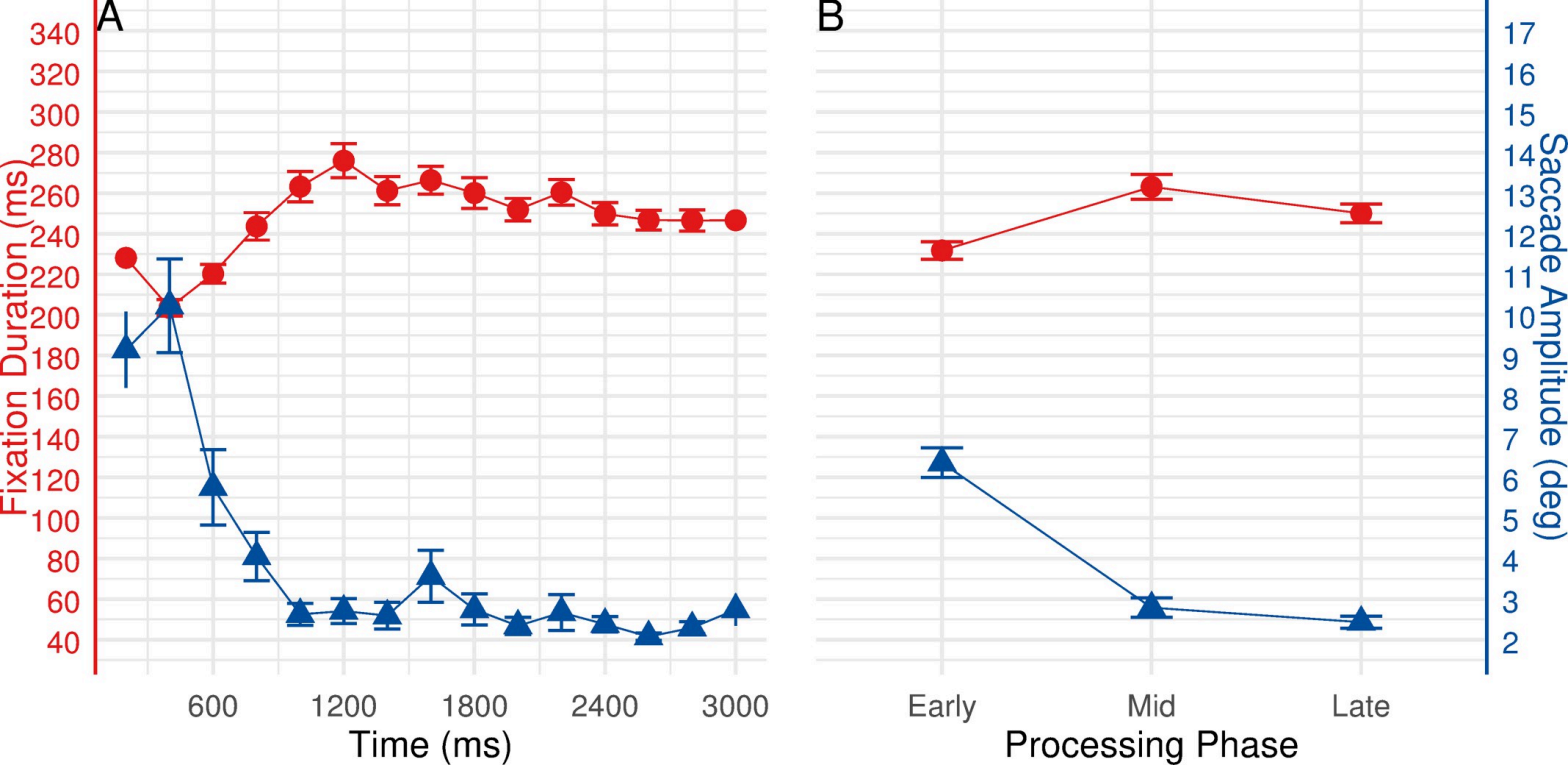
Eye movement behavior relative to the onset of new resource faces. Left panel (A) shows fixation durations (red line) and saccade amplitudes (blue line) over time. Right panel (B) shows estimated marginal means (estimated under the rm-ANOVAs) for fixation durations and saccade amplitudes during early, mid, and late phases. Error bars represent ±1 standard error.

In the analyses at the face level, fixation durations and saccade amplitudes after the onset of new resource faces were examined over a 3000 ms timescale (see Fig 5). While processing a partial alteration of the task environment (i.e., change in the resource area), fixation durations increased, whereas saccade amplitudes showed a steady decrease over the course of processing time (Fig 5A). The early, mid and late processing phases were classified by the same routine as described earlier. To determine whether eye movement patterns differed across processing phases, median values of fixation durations and saccade amplitudes were applied into rm-ANOVAs, respectively.

Analysis indicated that fixation durations varied significantly across processing phases, *F*(2, 108) = 27.356, *p* < 0.001, *η2* = 0.336. Follow-up post hoc pairwise t-test comparisons with Bonferroni correction revealed a significant increase in fixation durations from early to mid, as well as from early to late phase (both *ps* < 0.001, see Fig 5B): early phase (*M* = 231.73, *SE* = 4.34), mid phase (*M* = 263.10, *SE* = 6.05), and late phase (*M* = 250.02, *SE* = 4.55). Nevertheless, a significant decrease in fixation durations from mid to late phase was noted (*p* < 0.01). Furthermore, a significant main effect of processing phase on saccade amplitudes was found, *F*(1.59, 85.89) = 93.453, *p* < 0.001, *ε* = 0.795, *η2* = 0.634. The Bonferroni corrected post hoc pairwise t-test comparisons indicated that saccade amplitudes significantly decreased from early to mid, as well as from early to late phase (both *ps* < 0.001, see Fig 5B): early phase (*M* = 6.36, *SE* = 0.37), mid phase (*M* = 2.79, *SE* = 0.24), and late phase (*M* = 2.43, *SE* = 0.15).

### Fixation durations and saccade amplitudes at the area level

**Fig 6:**
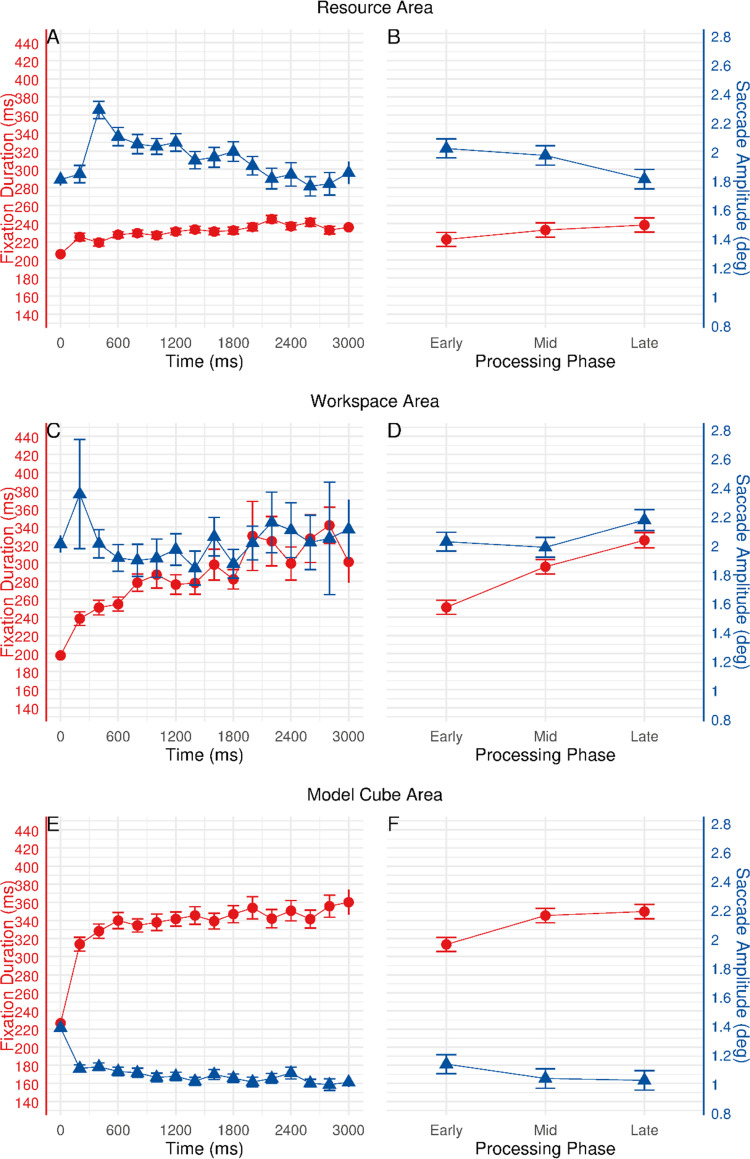
Eye movement behavior relative to the onset of eyes entering respective areas. Left panels (A, C, E) show fixation durations (red lines) and saccade amplitudes (blue lines) over time. Right panels (B, D, F) show estimated marginal means (estimated under linear mixed-effects models) for fixation durations and saccade amplitudes during early, mid, and late processing phases. Error bars represent ±1 standard error.

To investigate and compare eye movement behavior across different areas, fixation durations and saccade amplitudes within the first 3000 ms of entering an area were examined. The time course of eye movements when entering respective areas is depicted in Fig 6A, 6C and 6E). An increase in fixation durations was obtained for all three areas. Differences were also observed in the behavior of saccade amplitudes, with a decrease over time for resource and model cube areas. Only the workspace area revealed no consistent change in saccade amplitudes with time. To better identify the ambient to focal processing shifts, we further compared gaze patterns during early, mid, and late phases in respective areas (see Fig 6B, 6D and 6F). Early, mid, and late processing phases were classified following the same routines as described earlier.

As discussed earlier in the data analyses section, the eye movement data obtained for each of the areas were unbalanced and the data was not equally distributed with time (see Fig 2 and Table 1). Consequently, when fixation durations/saccade amplitudes were binned into 200 ms bins by calculating the median values, some observations were missing across time points from the data for workspace area (i.e., 9.7% data were missing, and 21 subjects had missing observations). In such an unbalanced case, linear mixed-effects models offer a more powerful and versatile framework for the analysis of unbalanced data than many other methods [32]. We, therefore, used linear mixed-effects models (lmerTest package) [33] to test the hypothesis that fixation durations and saccade amplitudes would show phasic changes over time but also differ across the three areas. Processing phase and area (with the interaction term) were entered into the models as fixed effects, subject was entered as a random effect, and median fixation durations/saccade amplitudes were entered as the dependent variables in separate mixed-effects models. In addition, the Kenward-Roger method was used for approximating the denominator degrees of freedom for tests of the fixed effects [34].

For fixation durations in different areas, significant effects were found for the main effects of processing phase, *F*(2, 108.17) = 55.242, *p* < 0.001, and area, *F*(2, 108.54) = 107.022, *p* < 0.001, but also the interaction between processing phase and area, *F*(4, 214.09) = 9.498, *p* < 0.001. Bonferroni corrected pairwise comparisons were subsequently performed to further investigate any differences between levels of the two factors (3 processing phases × 3 areas). Pairwise comparisons with area as moderator revealed a significant increase in fixation durations for all the areas (see Fig 6B, 6D and 6F). Specifically, in the resource area, fixation durations significantly increased from early to late processing phase (*p* < 0.05, see Fig 6B): early phase (*M* = 222.51, *SE* = 7.72), mid phase (*M* = 232.97, *SE* = 7.90), and late phase (*M* = 238.48, *SE* = 7.90). In the workspace area, fixation durations significantly increased throughout the three phases (all *ps* < 0.001, see Fig 6D): early phase (*M* = 251.01, *SE* = 7.73), mid phase (*M* = 295.89, *SE* = 8.03), and late phase (*M* = 325.17, *SE* = 8.38). In the model cube area, fixation durations significantly increased from early to mid, as well as from early to late phase (both *ps* < 0.001, see Fig 6F): early phase (*M* = 313.46, *SE* = 7.72), mid phase (*M* = 345.44, *SE* = 7.90), and late phase (*M* = 349.87, *SE* = 7.90). Meanwhile, pairwise comparisons with processing phase as moderator revealed significant differences between the areas. Fixations in the model cube area were significantly longer than in resource and workspace areas during all three phases (all *ps* < 0.05). In the workspace area, fixations were significantly longer than in the resource area during all three phases (all *ps* < 0.01).

For saccade amplitudes in different areas, the main effect of processing phase did not reach significance, *F*(2, 108.32) = 2.319, *p* = 0.103. Nevertheless, significant effects were found for the main effect of area, *F*(2, 108.88) = 143.816, *p* < 0.001, as well as for the interaction, *F*(4, 214.60) = 5.984, *p* < 0.001. Bonferroni corrected pairwise comparisons were subsequently performed to evaluate any differences between levels of the two factors (3 processing phases × 3 areas). Pairwise comparisons with area as moderator yield different results for different areas (see Fig 6B, 6D and 6F). In the resource area, saccade amplitudes significantly decreased from early to late phase (*p* < 0.001), as well as from mid to late phase (*p* = 0.015, see Fig 6B): early phase (*M* = 2.02, *SE* = 0.07), mid phase (*M* = 1.97, *SE* = 0.07), and late phase (*M* = 1.81, *SE* = 0.07). In contrast, saccade amplitudes in the workspace area significantly increased from early to late phase (*p* = 0.040), as well as from mid to late phase (*p* = 0.010, see Fig 6D): early phase (*M* = 2.02, *SE* = 0.07), mid phase (*M* = 1.98, *SE* = 0.07), and late phase (*M* = 2.17, *SE* = 0.07). In the model cube area, saccade amplitudes decreased by an average of 0.11 deg from early to late phase, but such a subtle decrease was reported to be insignificant (*p* = 0.113, see Fig 6F): early phase (*M* = 1.13, *SE* = 0.07), mid phase (*M* = 1.03, *SE* = 0.07), and late phase (*M* = 1.02, *SE* = 0.07). Moreover, pairwise comparisons with processing phase as moderator suggested that the saccades in the model cube area were significantly shorter than in resource and workspace areas during all three phases (all *ps* < 0.001). The comparison of saccade amplitudes between resource and workspace areas revealed a significant difference in the late processing phase (*p* < 0.001).

### The relationship between ambient to focal eye movement behavior and task performance

**Fig 7:**
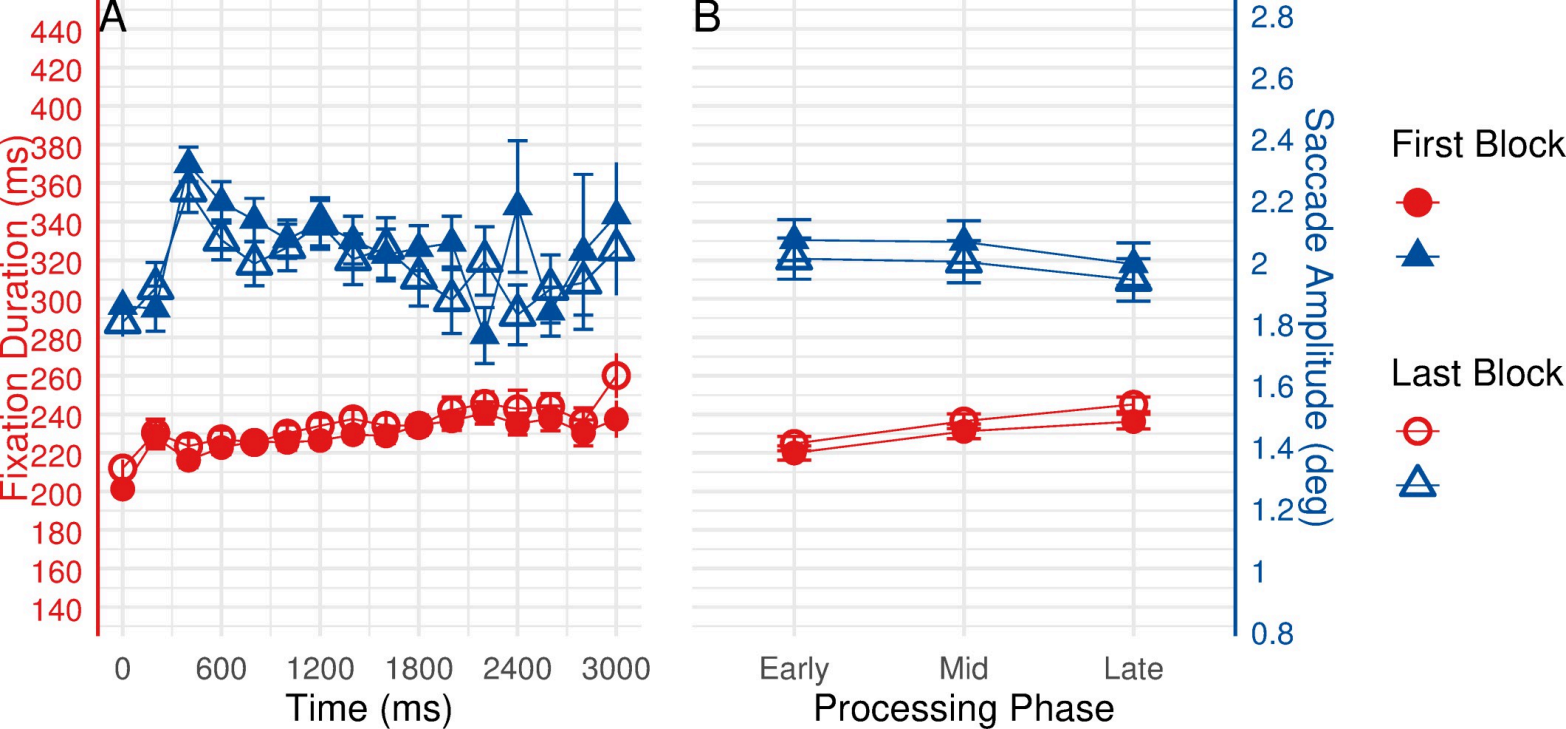
Eye movement behavior in resource area during the first and the last block. Left panel (A) shows time course of fixation durations (red lines) and saccade amplitudes (blue lines) in the resource area during the first and the last block. Right panel (B) shows early, mid, and late processing phases of fixation durations and saccade amplitudes (estimated marginal means, estimated under linear mixed-effects models) in the resource area during the first and the last block. Error bars represent ±1 standard error.

As the comparison of task solving time and error frequencies between the first (tasks 1–5) and the last (tasks 16–20) block revealed a significant improvement in subjects’ task performance, we speculated whether such an increase in expertise could result in an impact on ambient to focal behavior. The improved task performance could be considered as a general result of better performance in various sub-tasks. Because the clear indicators of ambient to focal processing (see Fig 6A and 6B) and a larger amount of gaze data (see Table 1) were obtained for the resource area, observations in resource area within the 3000 ms interval during the first and the last block (109,142 fixations and saccades) were subjected to comparison. The time course of eye movements when entering the resource area during the first and the last block is depicted in Fig 7A. An increase in fixation durations together with a decrease in saccade amplitudes were observed during both blocks. To statistically analyze the effect, we compared eye movement patterns among early, mid, and late processing phases in resource area between the two blocks to identify the ambient to focal processing shifts (see Fig 7B). The early, mid, and late processing phases were classified by the same routine as described earlier. Linear mixed-effects models [33] were used to test whether median fixation durations/saccade amplitudes would show phasic changes and be different during the first and the last block. The Kenward-Roger method [34] was used to estimate denominator degrees of freedom.

Analysis revealed significant differences of fixation durations between processing phases, *F*(2, 108.15) = 30.448, *p* < 0.001, as well as between the two blocks, *F*(1, 54.76) = 11.297, *p* = 0.001. The interaction between two factors was found to be insignificant, *F* < 1. Bonferroni corrected pairwise comparisons were subsequently performed to further investigate any differences between levels of the two factors (3 processing phases × 2 block). Pairwise comparisons with block as moderator revealed a significant increase in fixation durations during both blocks (see Fig 7B). Specifically, during the first block, fixation durations significantly increased from early to mid phase (*p* = 0.002), as well as from early to late phase (*p* < 0.001): early phase (*M* = 219.96, *SE* = 3.66), mid phase (*M* = 231.15, *SE* = 3.77), and late phase (*M* = 236.30, *SE* = 3.77). During the last block, fixation durations significantly increased throughout the three phases (all *ps* < 0.05): early phase (*M* = 224.82, *SE* = 3.66), mid phase (*M* = 236.47, *SE* = 3.77), and late phase (*M* = 245.16, *SE* = 3.79). Moreover, pairwise comparisons with processing phase as moderator showed that fixations in late processing phase during the last block were significantly longer than during the first block, *p* = 0.007.

Analysis of saccade amplitudes indicated no reliable effect of processing phase on saccade amplitudes, *F*(2, 108.11) = 1.576, *p* = 0.211. Moreover, no significant difference between the two blocks was reported, *F*(1, 54.51) = 2.100, *p* = 0.153. No interaction was obtained between two factors, *F* < 1. Nevertheless, examination of means suggested a decrease in saccade amplitudes over the three processing phases during the first and the last block (first block: *Ms* = 2.07 vs. 2.06 vs. 1.99, all *SEs* = 0.07; last block: *Ms* = 2.01 vs. 1.99 vs. 1.94, all *Ses* = 0.07, see Fig 7B).

### Task solving strategies

After completing RC tasks, subjects were required to answer two questions in the retrospective query: (i) Did you have a strategy for the RC task? Please describe the strategy you used. (ii) Please describe to what extent your strategy has changed during the experiment. All subjects reported that they employed certain strategies to accomplish the task. Their answers to the two retrospective questions were reviewed and categorized into six strategy features. However, the utilized strategies differed substantially across subjects. Thirty-nine (71%) subjects reported that they were focusing on the striking color patterns (of either resource or 3D model cube faces) to identify the target face for constructing the 2D copy. Twenty-three (42%) subjects found that manually rotating the 3D model cube to align the cube with patterns in either workspace or resource area helped reduce the complexity of the task. In order to understand the spatial logic of the 3D model cube, seventeen (31%) subjects focused on the center color of faces, while fifteen (27%) subjects focused on face edges and connections. Fifteen (27%) subjects claimed that they employed mental rotation or held copying-details in their mind while solving the task. Eight (15%) subjects commented that they were counting the numbers of different colors per face to identify the target face in resource area. In addition, thirty-nine (71%) subjects reported that their strategies formed and improved over time, whereas sixteen subjects (29%) did not change their strategies throughout the experiment.

We could not conclude a most common strategy for solving the RC task from subjects’ answers, since their descriptions differed in the way they included and sequenced the above-mentioned features. However, this result highlights one point that there appears to be no standard way for solving the RC task, and strategies vary widely in details subjects provide.

## Discussion

The present study investigated patterns of eye movements while subjects engaged in an active task, i.e., assembling a Rubik’s Cube, to gain further insights into the temporal dynamics between ambient and focal attention. In particular, we analyzed the time course of fixation durations/saccade amplitudes at multiple levels of a RC task (the task level, the face level, and the area level). Results of analyses at the task and the face level replicated and extended the previous findings [14,15,20,23] by showing that subjects shifted from ambient to focal visual processing following different levels of external changes to the stimuli (i.e., the onset of new task environment or a partial alternation of the task environment). More importantly, the results of analyses at the area level provide a new aspect that ambient to focal processing could also be identified when there were no external changes to the stimuli (task environment) but when internal representations of the task were hypothesized to change, such as switching between different sub-tasks. Furthermore, while diverse strategies were applied and a substantial improvement in behavioral performance was observed, the shifts between ambient and focal processing modes remain stable, suggesting that ambient and focal eye movement characteristics might serve as a probe for the attentional state in task processing.

While previous studies were rather focused on examining the temporal appearance of ambient and focal processing modes based on the trial onset design [14,15,19,20], the present study investigated eye movement behavior during processing different levels of external changes in a continuous task setting. In these previous studies, shifts from ambient to focal gaze pattern were observed after the onset of a new visual stimulus. Results of analyses at the task level replicated the previous findings indicating ambient to focal processing shifts after the onset of a new task. Moreover, while subjects perceived only a partial alternation of the visual environment (i.e., only one area in the task scenario changed), we observed a robust increase in fixation durations and a decrease in saccade amplitudes over the course of processing time. Results of analyses at the face level extended the previous findings to demonstrate that external changes in the visual environment always provoke the pronounced ambient to focal processing behavior.

Furthermore, the present study investigated whether subjects shift back and forth from ambient to focal processing also during the absence of external changes, i.e., when only internal processes can trigger such a behavior. In order to solve the RC task, subjects had to switch between different sub-tasks. Each sub-task was performed in respective spatial areas of the task scenario. Analyzing eye movement behavior across the three areas revealed similar temporal changes in resource and model cube areas: Within the first 3000 ms of entering the respective areas, fixation durations gradually increased while at the same time saccade amplitudes decreased. This finding adds significantly to existing knowledge in the way that ambient to focal behavior can also be evoked by internal triggers or mental shifts between different sub-tasks. However, clear evidence was found for the resource area and the model cube area, but this was only partly the case for the workspace area: No ambient to focal behavior was identified for saccade amplitudes. The unique gaze pattern in the workspace area might potentially stem from the low cognitive load and the recognition of performance errors. In the workspace area, subjects had to comprehend the spatial connections between the previously completed faces of the 2D pattern in order to determine the position in which to place the selected resource face. It might become easier as the workspace area fills up over the course of the task. During arranging the selected face in the workspace area, if subjects made an error, the selected face was always restored to its former location from the workspace to the resource area. Concurrently, subjects processed information about their performance error in the workspace, and such occurrence of an error led them to find the solution from the other two areas. Moreover, the less clear behavior of saccade amplitudes might also be attributable to the relatively small amount of data gathered in the workspace area (Table 1; also see the error bars in Fig 6C and 6D).

In addition to the temporal changes, the comparison of fixation durations and saccade amplitudes revealed substantial differences in eye movement behavior across the three areas. The distinct eye movement behavior could be explained as an adjustment of oculomotor behavior in accordance with the specific sub-task demands/characteristics in respective areas. Comparing the resource area to the workspace and model cube areas, subjects applied larger saccades and shorter fixations as the optimal strategy for selective searching in the resource area [35,36]. To rapidly sample information from the eight resource faces, subjects had to actively make saccades from one face to another. Such visual search strategy facilitated accessing information from a greater area of the visual field, which could help to improve searching performance [37]. On the contrary, overall longer fixations and shorter saccades were observed in the model cube area. Comparing to the resource and workspace areas, the model cube area contained a stationary salient configuration (3D model cube) which exhibited a much smaller attentional window. Subjects were encouraged to place an emphasis on information gathering about the model configuration. Correspondingly, subjects adjusted their perceptual strategy by applying longer fixations and shorter saccades to access more information from each fixation while actively and manually rotating the model cube. Moreover, when subjects rotated the model cube, it was likely that they generated small-amplitude smooth pursuits to follow some distinct features of the model cube, which might also lead to longer fixations [38]. Our results are consistent with the findings from earlier work [36]; different gaze behavior was employed rationally based on the demand of the ongoing sub-task. Moreover, previous research suggested that different oculomotor behaviors were engaged in different display types and task demands, indicating that fixation- and saccade-related metrics changed as a function of visual scanning strategy [39–41].

The distinct eye movement behaviors observed in different areas are not pure measures of the correlates of respective sub-tasks processing; they also reflect the differences in spatial dimension and the number of objects covered by each area. For instance, the differences in spatial dimensions between the three areas could partially account for the systematic variations in the number of fixations allocated to each area (see Fig 2): As the resource area covered the largest portion of the task scenario, a greater number of fixations were naturally allocated there. On the contrary, as the workspace area gradually filled up over the course of the task, fewer fixations were required for scanning through the available positions during the later stage of the task. Moreover, it is conceivable that fixation durations and saccade amplitudes varied along with the number of objects exhibited in each of the areas: As discussed above, more available objects in the resource area increased the size of the attentional window and demanded longer saccades to locate the intended object. In contrast, the reference configuration in the model cube area was smaller in size but denser with information (e.g., the spatial logic of the 3D model cube), where subjects appeared to be able to process more information per fixation than in areas with low information density (i.e., resource area or workspace area), and fixations were thereby prolonged to make the appropriate processing possible [42]. Even so, regardless of the distinct sub-tasks imposed in each of the areas or the spatial differences between these areas, a general pattern of increasing fixation durations and decreasing saccade amplitudes with time was replicated across resource and model cube areas. These results are consistent with Unema et al.’s [15] and Pannasch et al.’s [14] findings; although both fixation duration and saccade amplitude are affected accordingly by the number of objects in a scene, the ambient and focal characteristics in eye movements for early and late phases of viewing remain stable.

Taken together, results of the analyses at the area level provide support for our hypothesis that internal triggers have a similar influence on attentional processing—at least as it is evidenced in eye movement parameters—as external events. More importantly, area specific sub-tasks do not appear to influence the occurrence of ambient and focal eye movement characteristics in the time course of processing. While substantial differences in fixation and saccades were observed, shift from ambient to focal processing modes were found consistent between areas.

In addition, it is worth noting that the 3000 ms analysis window used in the present study for measuring ambient/focal gaze behavior was much smaller when compared to the analysis windows that used in previous research [14,15,23]. For instance, in Pannasch et al.’s [14] study, the ambient and focal gaze patterns were investigated during different viewing conditions across an analysis window of 6 seconds. Whereas in Unema et al.’s [15] and Eisenberg et al.’s [23] studies, the ambient/focal processing shifts were examined within a 20 second analysis window. In these scene viewing studies, presentation time of visual stimuli could be well-controlled. However, in the present study, subjects shifted their gaze between different areas of the task scenario in a self-paced manner. The 3000 ms analysis window was chosen based on the distribution of gaze data over time, which has only considered the context of the RC task. Whereas in real-life task performance, people might sustain the voluntary deployment of attention to a certain region for a longer time. Therefore, the generalizability of this analysis window size to other settings may be limited. Continued focus on how to determine an analysis window which is representative of the entire data could be useful for further research.

Over time, the performance of the task became mastered to a certain extent and the complexity of the task became less apparent. By examining the task solving time and accuracy throughout the experiment, we found indications of increasing expertise. Compared to the first block (tasks 1–5), subjects required less time to solve one task and made fewer errors during the last block (tasks 16–20). In addition, the assessment of strategies subjects described in the retrospective query suggested that subjects differed widely in the way they solve the RC task.

The comparison of eye movement patterns between the first and the last block provides insights into the linkages between ambient to focal attention and behavioral performance. Regardless of the significantly improved task performance, fixation durations and saccade amplitudes in the resources area during the two blocks showed similar phasic changes within an interval of 3000 ms: Fixation durations increased while saccade amplitudes decreased throughout the three processing phases (early, mid, and late), indicating shifts from ambient to focal processing mode. Such shifts in visual processing modes appeared to be independent of task performance. This result suggests that ambient to focal gaze behavior might serve as a probe for the attentional state in task processing, independently of individual task proficiency.

Overall, the present study reported the time course of eye movement parameters (fixation durations and saccade amplitudes) as a continuous measure of visual processing as the task progresses. The results suggest that the initial ambient gaze pattern is followed by a switch to more focal viewing across multiple levels of task processing. Such ambient to focal processing shifts were not only identified after the onset of external changes in the environment, they also occurred when switching between sub-tasks while no external event was given. Interestingly, a comparison of ambient to focal processing in response to external changes (new task/new resource faces) versus internal changes (switching between sub-tasks) reveals noticeable differences in fixation- and saccade-related metrics (cf. x-axis values of fixation duration and saccade amplitude in Figs 4–6). Especially during the early (ambient) phases, the length of saccades after the onset of external changes were much longer than after the internal changes. Apart from the fact that analyses of ambient to focal processing as a result of external changes (at the task and the face level) were based on the entire task scenario instead of individual sub-areas, the external changes modify features of the task environment (even at the face level, the largest portion of the task scenario—the resource area—changes), comprehending such external events requires subjects to update their working memory representations of the current environment. It is sensible that the longer saccades would best allow for gathering as much information as possible about the general context of the task to enable updating of working memory. In general, our findings add further evidence to the existence of ambient and focal processing mechanisms in continuous and more complex task settings.

However, the reported ambient to focal behaviors at the area level were observed following the onset of the subject’s eyes entering a new area, suggesting that spontaneous visual exploration was a contributing factor in eliciting a shift from ambient to focal processing. It remains open for further research whether a sudden change of task demand/characteristic would evoke ambient gaze behavior for exploratory processing. Moreover, a further investigation is required to address how different visual features can potentially affect the ambient to focal processing strategy. It would be interesting to see whether people would be automatically biased towards ambient processing (e.g., they may take longer to reach the focal mode) when perceiving a number of objects scattered over a space, and whether they would be biased towards focal processing (e.g., reach the focal mode sooner) when perceiving a single salient object. Finally, given that the different areas were fixed across subjects, with the resource and workspace features to the left and the model cube to the right, it is possible that such stimulus design may result in left-right saccade asymmetries, which in turn could affect our observations. In previous studies [43–45], left-right saccade asymmetries have been observed when visual stimuli were presented in the peripheral visual field, in which some subjects exhibited a directional preference to the left or to the right in their saccade responses. This question could be addressed in future work by changing the layout of the task scenario to systematically examine whether cognitive factors, such as visuo-spatial attentional bias, may play a role in saccade behavior.

## Conclusions

The present study provides not only updated evidence that a shifting relationship between ambient and focal processing modes is manifested in eye movement behavior; ambient to focal processing works as the basic attentional mechanism in task processing. The combination of subjects’ eye movement patterns, task performance, and task solving strategy descriptions allowed us to open new perspectives for the investigation of complex task processing, also for the development of attention-sensitive interfaces. Future work should therefore focus on more applied settings.

## Supporting information

S1 Video Video recording of one RC task. A video recording of one RC task is available online: https://doi.org/10.17605/OSF.IO/WRV37.
(DOCX)

S1 Data Complete data are available online: https://doi.org/10.17605/OSF.IO/CQSJG. (DOCX)

S1 Text Partially overlapping data between the task/face level and the area level. (DOCX)
