# Supplementary material for: Eye movement patterns in complex tasks: Characteristics of ambient and focal processing
Source: PLoS One. 2022 Nov 9;17(11):e0277099. doi: 10.1371/journal.pone.0277099 (PMC9645626; doi:10.1371/journal.pone.0277099)
Supplement: S1 Video — A video recording of one RC task is available online: https://doi.org/10.17605/OSF.IO/WRV37. (DOCX) [file pone.0277099.s001.docx]

**S1 Video. Video recording of one RC task.** A video recording of one RC task is available online: https://doi.org/10.17605/OSF.IO/WRV37.
