# Supplementary material for: Eye movement patterns in complex tasks: Characteristics of ambient and focal processing
Source: PLoS One. 2022 Nov 9;17(11):e0277099. doi: 10.1371/journal.pone.0277099 (PMC9645626; doi:10.1371/journal.pone.0277099)
Supplement: S1 Data — (DOCX) [file pone.0277099.s002.docx]

**S1 Data. Complete data are available online: https://doi.org/10.17605/OSF.IO/CQSJG.**
