# Supplementary material for: Eye movement patterns in complex tasks: Characteristics of ambient and focal processing
Source: PLoS One. 2022 Nov 9;17(11):e0277099. doi: 10.1371/journal.pone.0277099 (PMC9645626; doi:10.1371/journal.pone.0277099)
Supplement: S1 Text — (DOCX) [file pone.0277099.s003.docx]

**S1 Text. Partially Overlapping Data Between the Task/Face Level and the Area Level.**

It should be noted that the data pre-processing procedures described in the data analyses section allowed fixations/saccades to be counted in multiple roles while formulating different time courses of processing. For instance, a fixation that occurred 2000 ms after the onset of the task, would be naturally allocated into the late phase of processing at the task level. Meanwhile, this fixation might also be the first fixation that happened after subject’s eyes entered the resource area, which would be assigned into the early phase of processing at the area level. As a result, when analyzing the time course of eye movements across different levels of processing, some fixations/saccades might be applied parallel to both early and late phases in respective calculations. To minimize the ambiguity in assigning the same gaze activity to different time phases, the following steps were taken: (i) We detected a total of 38,486 fixations and saccades that played roles in both task/face level processing and area level processing. (ii) Considering the dataset for analyses at the area level was much larger in the present study, we opted to remove the temporal observations that contained the above overlapping fixations/saccades from the dataset prior to area level analyses. Correspondingly, all fixations and saccades would only be counted once in the analyses at either task/face level or area level. This left a total of 347,642 fixations and saccades as pre-processed data for area level analyses. (iii) We then analyzed the time course of eye movements across different areas using the same methods as described in the results section. When comparing the results of area level analyses with and without the overlapping gaze activities, we found that eye movement patterns over courses of processing were not altered substantially by excluding the overlapping fixations/saccades. Moreover, the statistical significance of the findings was qualitatively similar. As demonstrated above, allowing fixations/saccades to play multiple roles during different levels of processing had minimal impact on overall results. We therefore chose to stick with the approach that was more closely related to the primary data (further information about the data and additional analyses are available online: <https://doi.org/10.17605/OSF.IO/FHD7S>).
